# Prolonged venous transit is associated with lower odds of excellent recovery after reperfusion in anterior large‐vessel occlusion stroke

**DOI:** 10.1111/ene.16563

**Published:** 2024-12-02

**Authors:** Hamza Adel Salim, Dhairya A. Lakhani, Janet Mei, Licia Luna, Mona Shahriari, Nathan Z. Hyson, Francis Deng, Adam A. Dmytriw, Adrien Guenego, Victor C. Urrutia, Elisabeth B. Marsh, Hanzhang Lu, Risheng Xu, Rich Leigh, Dylan Wolman, Gaurang Shah, Benjamin Pulli, Gregory W. Albers, Argye E. Hillis, Rafael Llinas, Kambiz Nael, Max Wintermark, Jeremy J. Heit, Tobias D. Faizy, Vivek Yedavalli

**Affiliations:** ^1^ Department of Radiology, Division of Neuroradiology Johns Hopkins Medical Center Baltimore Maryland USA; ^2^ Department of Neuroradiology MD Anderson Medical Center Houston Texas USA; ^3^ Department of Radiology West Virginia University Morgantown West Virginia USA; ^4^ Neuroendovascular Program Massachusetts General Hospital, Harvard University Boston Massachusetts USA; ^5^ Neurovascular Centre, Department of Medical Imaging and Neurosurgery St. Michael's Hospital Toronto Ontario Canada; ^6^ Department of Diagnostic and Interventional Neuroradiology Erasme University Hospital Brussels Belgium; ^7^ Department of Radiology Brown University Providence Rhode Island USA; ^8^ Department of Radiology University of Michigan Ann Arbor Michigan USA; ^9^ Department of Interventional Neuroradiology Stanford Medical Center Palo Alto California USA; ^10^ Department of Radiology & Biomedical Imaging University of California San Francisco California USA; ^11^ Department of Radiology, Neuroendovascular Program University Medical Center Münster Germany

**Keywords:** Acute ischemic stroke (AIS), Computed tomography perfusion (CTP), Intravenous thrombolysis (IVT), Large‐vessel occlusion (LVO), Mechanical thrombectomy (MT), Prolonged venous transit (PVT), Venous outflow (VO)

## Abstract

**Background and purpose:**

Acute ischemic stroke due to anterior circulation large‐vessel occlusion (AIS‐LVO) remains a leading cause of disability despite successful reperfusion therapies. Prolonged venous transit (PVT) has emerged as a potential prognostic imaging biomarker in AIS‐LVO. We aimed to investigate whether PVT is associated with a decreased likelihood of excellent functional outcome (modified Rankin Scale [mRS] score of 0–1 at 90 days) after successful reperfusion.

**Methods:**

In our prospectively collected, retrospectively reviewed database, we analyzed data from 104 patients with AIS‐LVO who achieved successful reperfusion (modified Thrombolysis in Cerebral Infarction score of 2b/2c/3) between September 2017 and September 2022. PVT was defined as a time to maximum (Tmax) of ≥10 s in the superior sagittal sinus and/or torcula on computed tomography perfusion (CTP) imaging. Patients were categorized into PVT‐positive (PVT+) and PVT‐negative (PVT–) groups. The primary outcome was excellent functional recovery at 90 days.

**Results:**

Of the 104 patients, 30 (29%) were PVT+. Excellent functional outcome was achieved in 38 patients (37%). PVT+ patients had a significantly lower rate of excellent recovery compared to PVT– patients (11% vs. 39%; *p* < 0.001). After adjusting for possible confounders, PVT positivity was independently associated with lower odds of excellent recovery (adjusted odds ratio 0.11, 95% confidence interval 0.02 to 0.48; *p* = 0.006).

**Conclusions:**

Among patients with AIS‐LVO who achieved successful reperfusion, PVT positivity was independently associated with a decreased likelihood of excellent functional outcome at 90 days. Assessment of PVT on CTP may provide valuable prognostic information and aid in clinical decision making for patients with AIS‐LVO.

## INTRODUCTION

Acute ischemic stroke due to large‐vessel occlusion (AIS‐LVO) in the anterior circulation remains a significant cause of morbidity and mortality [1]. Despite advances in reperfusion therapies, such as mechanical thrombectomy (MT) [2, 3] and its association with improved outcomes, variability in functional recovery persists among patients, even when successful reperfusion is achieved. Excellent functional recovery, defined as a modified Rankin Scale (mRS) score of 0 to 1 at 90 days, represents a key clinical endpoint, as it correlates with a return to pre‐stroke levels of independence and quality of life.

Venous outflow (VO) is attracting increasing attention as a prognostic imaging biomarker in patients with AIS‐LVO. Patients who present with AIS‐LVO have recently had their VO profiles evaluated as part of the cerebral collateral cascade [4, 5, 6, 7, 8]. Despite successful reperfusion, unfavorable VO has been identified as a predictor of post‐procedural cerebral edema, edema progression, hemorrhagic transformation, and poor clinical outcomes [7, 9, 10, 11]. In contrast, patients treated with MT are more likely to experience favorable clinical outcomes when their VO is favorable [9].

The use of computed tomography perfusion (CTP) is common in several medical facilities for the purpose of triaging patients with AIS‐LVO for MT [12, 13]. Given the abundant data obtained from multiphase acquisition, CTP can also offer potentially valuable insights into VO. Therefore, only a small number of studies have specifically examined the effectiveness of CTP in evaluating VO for patients with AIS‐LVO [14, 15, 16]. Prolonged venous transit (PVT), defined as the presence of time to maximum (Tmax) ≥10 s in at least one of the major dural venous sinus locations (i.e., superior sagittal sinus [SSS] and/or torcula), is thought to represent a surrogate of VO at the level of the proximal dural venous sinuses and is easily interpreted visually using post‐processed Tmax maps on CTP. This binary marker has demonstrated its utility by revealing an association with 90‐day mortality in successfully reperfused AIS‐LVO patients [17]. However, its prognostic significance in relation to excellent functional outcome (90‐day mRS score 0–1) remains unexplored [18, 19].

In this study, we investigated the relationship between PVT and excellent functional outcome (mRS score 0–1) at 90 days. We hypothesized that PVT positivity would be associated with a decreased likelihood of excellent functional outcome at 90 days compared to PVT negativity.

## METHODS

Deidentified data will be made available to qualified investigators upon reasonable request to the corresponding author. This retrospective study was approved by the institutional review board, with waiver of informed consent (JHU‐IRB00269637) [12, 20, 21, 22, 23, 24, 25]. This observational study followed the Strengthening the Reporting of Observational Studies in Epidemiology (STROBE) checklist guidelines.

### Population

From a prospective registry of patients with acute ischemic stroke between September 1, 2017 and September 22, 2022, we retrospectively analyzed patients meeting our inclusion criteria. Inclusion criteria included (i) confirmed anterior circulation large‐vessel occlusion on computed tomography angiography (CTA) using baseline comprehensive computed tomography (CT) evaluation, inclusive of noncontrast CT, CTA, and CTP [2]; successful reperfusion with intravenous thrombolysis (IVT) or MT, defined as modified Thrombolysis in Cerebral Infarction (mTICI) score of 2b/2c/3; and [3] available 90‐day mRS score. Patients who were not successfully reperfused despite MT attempts, or who lacked 90‐day follow‐up data, were excluded from the analysis.

### Clinical data collection

Baseline clinical data were ascertained by certified neurologists or nurse practitioners at the time of the clinical encounter and were retrospectively extracted from the electronic medical record. These parameters included demographics, risk factors for acute ischemic stroke (including diabetes, hypertension, coronary artery disease, and atrial fibrillation), prior stroke history, antiplatelet or anticoagulant use, tobacco use, alcohol use, admission glucose, premorbid mRS score and admission National Institutes of Health Stroke Scale (NIHSS) score.

### Clinical outcomes assessment

The 90‐day mRS scores were calculated by certified neurologists or nurse practitioners.

### Interventional data collection

Per institutional protocols, the decision to treat with either IVT or MT is made by multidisciplinary stroke team consensus. If the patient was treated with MT, thrombectomy technique (i.e., aspiration only, stent retriever only, or combination) and devices used were per discretion of the neurointerventionalist. Last‐known‐well‐to‐door and door‐to‐recanalization times were collected. The final post‐treatment mTICI score was determined by the treating neurointerventionalist and verified retrospectively based on two‐expert consensus review.

### Imaging data collection

Imaging data obtained from contemporaneous radiology reports were retrospectively reexamined and validated by an experienced neuroradiologist. Postprocessed or otherwise retrospectively evaluated imaging data were obtained by an experienced neuroradiologist.

Alberta Stroke Program Early CT scores (ASPECTSs) were calculated from pretreatment noncontrast CT by an experienced neuroradiologist (V.Y.; 9 years of experience). Presence of large‐vessel occlusion, segment of occlusion, and laterality of occlusion were identified on the basis of baseline CTA by an experienced neuroradiologist (V.Y.). Collateral status assessments were performed on single‐phase CTA as established by Tan et al. [26, 27]. The presence of complications such as hemorrhagic transformation, as defined by the ECASS 2 (European Cooperative Acute Stroke Study) trial [28, 29], was assessed on follow‐up noncontrast CT or susceptibility‐weighted magnetic resonance imaging within 48 h of initial presentation.

### CTP parameters

Whole‐brain pretreatment CTP was performed on the Siemens Somatom Force (Erlangen, Germany) with the following parameters: 70 kVP, 200 effective mAs, rotation time 0.25 s, average acquisition time 60 s, collimation 48 × 1.2 mm, pitch value 0.7, and four‐dimensional range 114 mm × 1.5 s. Pretreatment CTP images were then postprocessed using RAPID commercial software (IschemaView, Menlo Park, CA, USA) to generate quantitative relative cerebral blood flow (rCBF) volumes, Tmax volumes, and qualitative Tmax maps [30].

### PVT assessment

**FIGURE 1:**
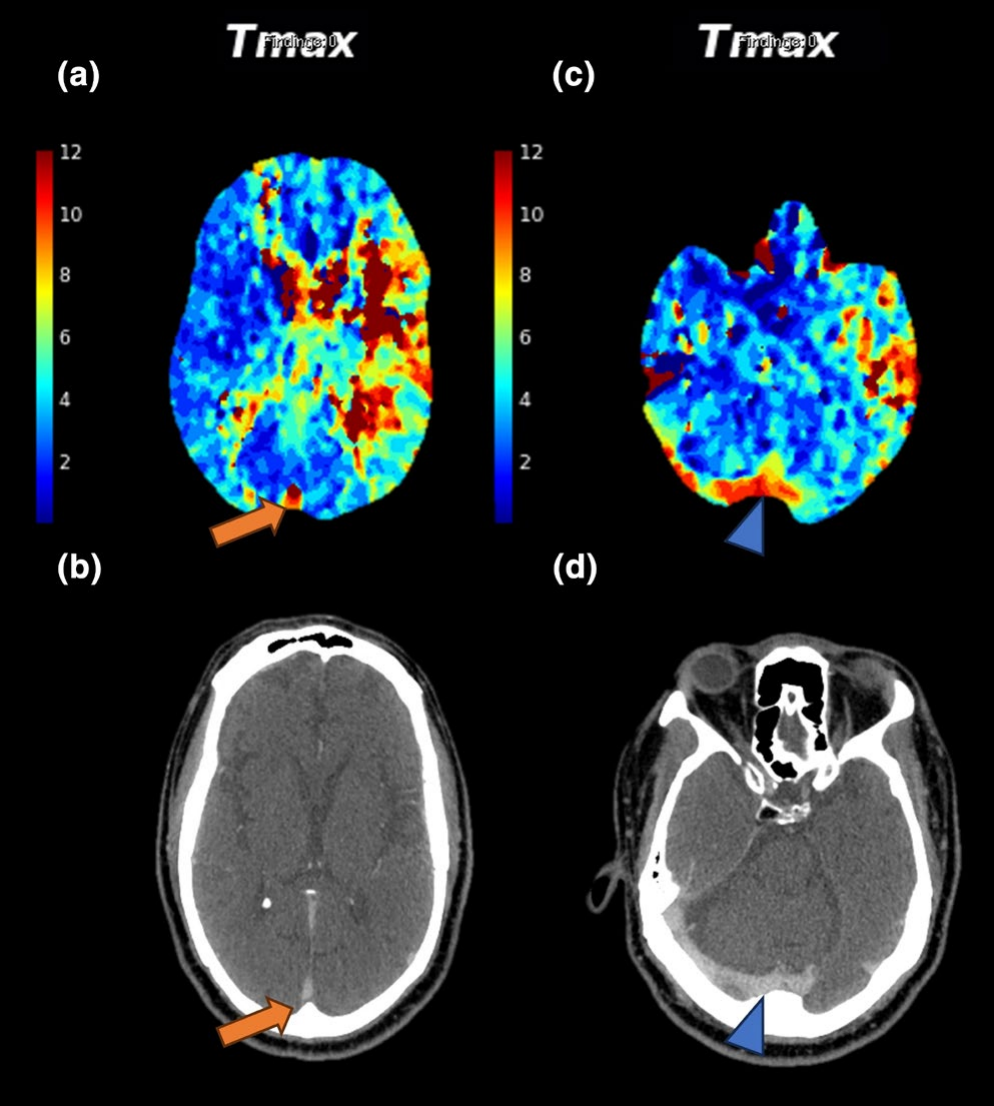
Imaging from a 68‐year‐old man presenting with left M1 occlusion and aphasia. His admission National Institutes of Health Stroke Scale score was 24. Post‐thrombectomy achieved modified Thrombolysis in Cerebral Infarction score 2b reperfusion after one pass. The 90‐day modified Rankin Scale score was 3. Top row: Tmax perfusion maps demonstrating prolonged venous transit in (a) the posterior superior sagittal sinus (orange arrow) and (c) the torcula (blue arrowhead). Bottom row: (b) and (d) corresponding computed tomography angiography images highlighting the same venous structures (arrows).

**FIGURE 2:**
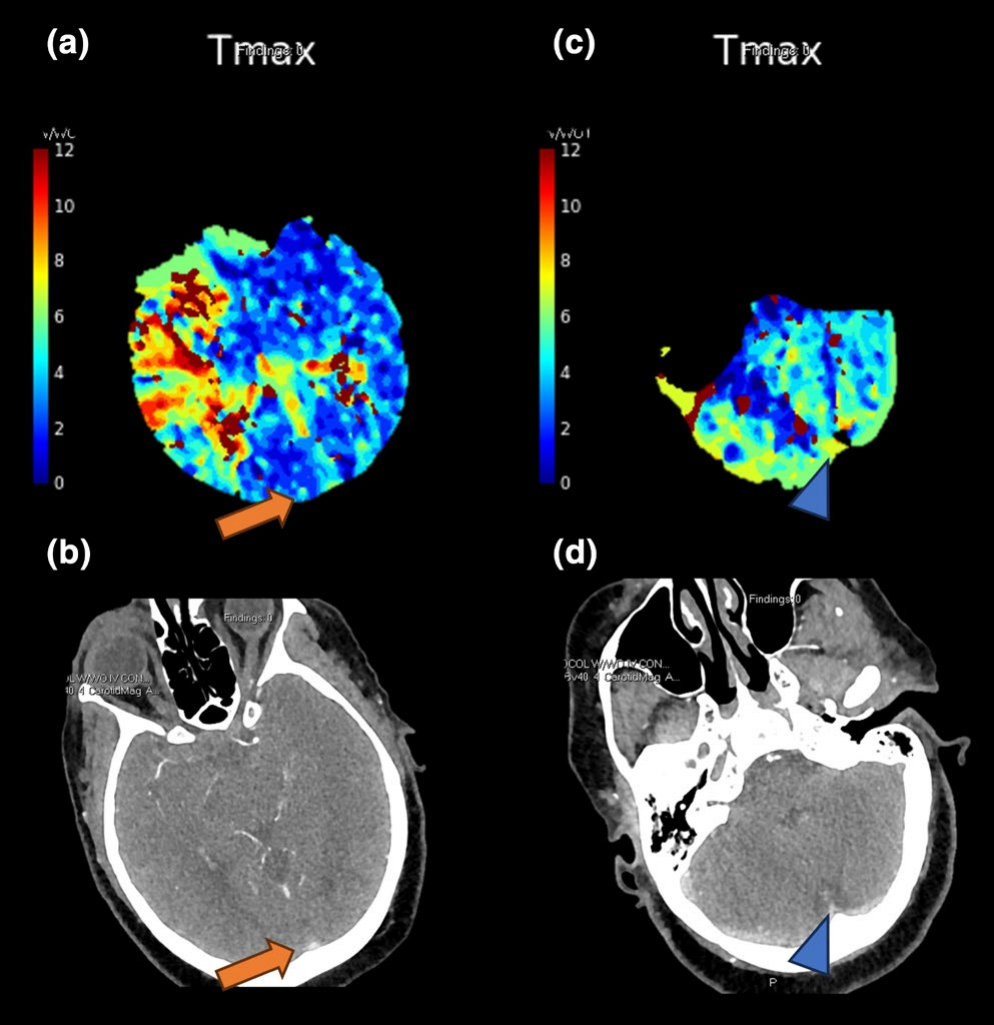
Imaging from a 72‐year‐old woman presenting with right proximal M1 occlusion and left extremity weakness. Her admission National Institutes of Health Stroke Scale score was 22. Post‐thrombectomy achieved modified Thrombolysis in Cerebral Infarction score 2c reperfusion after one pass. The 90‐day modified Rankin Scale score was 0. Top row: Tmax perfusion maps showing no prolonged venous transit in (a) the posterior superior sagittal sinus (orange arrow) or (c) the torcula (blue arrowhead). Bottom row: (b) and (d) corresponding computed tomography angiography images highlighting the venous structures (arrows).

Based on qualitative Tmax maps, PVT was assessed within the posterior SSS at the level of the lateral ventricle occipital horns to incorporate more proximal venous drainage as well as at the torcula to account for deep venous drainage patterns. PVT positivity was defined as a Tmax ≥10 s within the posterior SSS, torcula, or both areas when using the timing key adjacent to the image. PVT assessments were performed by a board‐certified neuroradiologist (V.Y., 10 years of working experience) and a diagnostic neuroradiology fellow (D.A.L., 4 years of working experience), blinded to the clinical information. Discrepancies were resolved by consensus review. Exemplar patients with PVT‐positive (PVT+) and PVT‐negative (PVT−) profiles are shown in Figures 1 and 2, respectively.

### Outcome measures

The primary outcome was excellent clinical recovery, defined as a 90‐day mRS score of 0 or 1.

### Statistical analysis

The objective of this study was to assess the association between PVT positivity and excellent clinical outcome, defined as a 90‐day mRS score of 0 or 1. Descriptive statistics were used to summarize the data first. Continuous data are reported as median and interquartile range (IQR) and were compared using Wilcoxon rank‐sum tests. Categorical variables are reported as frequencies and were compared using chi‐squared tests or Fisher's exact test, where cell count was <5. Univariable logistic regression was used to identify potential variables associated with excellent outcome, as defined by a *p* value of <0.1, and these were then included in the subsequent multivariable logistic regression model. Forced inclusion of some key variables in the initial model was carried out based on scientific rationale and their known clinical significance. Stepwise backward selection was used, maintaining variables in the model only if they met the retention threshold of a *p* value <0.1. Statistical significance was set at a *p* value of < 0.05. The results of the logistic regression analyses are expressed as odds ratios (ORs) with 95% confidence intervals (CIs). All statistical analyses were conducted using R software (version 4.2.2.) [31].

## RESULTS

### Baseline characteristics

A total of 104 patients with AIS‐LVO were included in the study. The median (IQR) age of the population was 70 (62–79) years, with 56% being female. Patients were stratified into two groups PVT+ (*n* = 30) or PVT– (*n* = 74). Baseline characteristics, including demographics, stroke risk factors, and clinical parameters, were similar in the two groups, with the exception of a significantly higher median admission NIHSS score in the PVT+ group (16 vs. 14; *p* = 0.038 [Table 1]).

**TABLE 1:** Baseline characteristics compared between patients with and without prolonged venous transit.

Variable	Overall, *N* = 104	PVT– group	PVT+ group	*p* ^a^
		*N* = 74	*N* = 30	
Age, median (IQR), years	70 (62, 79)	70 (61, 78)	72 (63, 82)	0.42
Sex, *n* (%)				
Female	58 (56)	45 (61)	13 (43)	0.1
Male	46 (44)	29 (39)	17 (57)	
Occlusion segment, *n* (%)				
ICA	8 (7.7)	4 (5.4)	4 (13)	0.38
M1	76 (73)	56 (76)	20 (67)	
Poximal‐M2	20 (19)	14 (19)	6 (20)	
Smoking status, *n* (%)	54 (52)	36 (49)	18 (60)	0.32
Alcohol use, *n* (%)	32 (31)	26 (36)	6 (20)	0.12
Hypertension, *n* (%)	83 (80)	56 (76)	27 (90)	0.1
Dyslipidemia, *n* (%)	55 (53)	39 (53)	16 (53)	0.95
Diabetes, *n* (%)	29 (28)	19 (26)	10 (33)	0.43
Heart disease, *n* (%)	52 (50)	38 (51)	14 (47)	0.67
Atrial fibrillation, *n* (%)	44 (42)	33 (45)	11 (37)	0.46
History of stroke/TIA, *n* (%)	17 (16)	12 (16)	5 (17)	>0.99
Chronic kidney disease, *n* (%)	20 (19)	15 (20)	5 (17)	0.67
Sleep apnea, *n* (%)	11 (11)	8 (11)	3 (10)	>0.99
Admission NIHSS score, median (IQR)	15 (11, 20)	14 (10, 19)	16 (14, 20)	0.038
Premorbid mRS score, *n* (%)				
0	83 (81)	57 (78)	26 (87)	0.32
1	20 (19)	16 (22)	4 (13)	
Stroke etiology (TOAST criteria), *n* (%)				
Large artery atherosclerosis	14 (14)	12 (16)	2 (6.7)	0.54
Cardioembolism	60 (58)	40 (55)	20 (67)	
Stroke of other determined etiology	3 (2.9)	2 (2.7)	1 (3.3)	
Stroke of undetermined etiology	26 (25)	19 (26)	7 (23)	
Occlusion laterality, *n* (%)				
Left	45 (50)	31 (47)	14 (58)	0.34
Right	45 (50)	35 (53)	10 (42)	

Abbreviations: ICA, internal carotid artery; IQR, interquartile range; mRS, modified Rankin Scale; NIHSS, National Institutes of Health Stroke Scale; PVT, prolonged venous transit; TIA, transient ischemic attack; TOAST, Trial of ORG 10172 in Acute Stroke Treatment.

^a^ Wilcoxon rank sum test; Pearson's Chi‐squared test; Fisher's exact test.

### Imaging and interventional data

Preintervention imaging demonstrated a higher median mismatch volume in PVT+ patients (118 [IQR 86–170] mL) compared to PVT– patients (81 [IQR 46–118] mL; *p* < 0.001). Other imaging parameters, including ASPECTS, hypoperfusion intensity ratio, and rCBF <20%, were not significantly different between groups (Table 2).

**TABLE 2:** Preintervention imaging, interventional, and postinterventional outcome parameters compared between patients with and without prolonged venous transit.

Variable	Overall, *N* = 104	PVT– group	PVT+ group	*p* ^a^
		*N* = 74	*N* = 30	
ASPECTS, median (IQR)	9.00 (7.75, 10.00)	9.00 (8.00, 10.00)	10.00 (7.25, 10.00)	0.67
rCBF <20% volume, median (IQR), mL	0 (0, 8)	0 (0, 8)	0 (0, 11)	0.22
Mismatch volume, median (IQR), mL	93 (57, 133)	81 (46, 118)	118 (86, 170)	<0.001
Mismatch ratio, median (IQR)	3.9 (2.8, 7.4)	3.9 (2.5, 7.2)	4.4 (3.4, 7.9)	0.6
Hypoperfusion intensity ratio, median (IQR)	0.40 (0.20, 0.50)	0.35 (0.20, 0.50)	0.45 (0.30, 0.50)	0.18
CBV index, median (IQR)	0.80 (0.70, 0.90)	0.80 (0.70, 0.90)	0.80 (0.70, 0.90)	0.8
PVT, *n* (%)	30 (29)	0 (0)	30 (100)	<0.001
Tan score (0–3), median (IQR)	2.00 (1.00, 2.00)	2.00 (1.00, 2.00)	2.00 (1.00, 2.00)	0.089
IVT administered, *n* (%)	32 (31)	24 (32)	8 (27)	0.56
MT attempted, *n* (%)	104 (100)	74 (100)	30 (100)	
Symptom onset to door time, median (IQR) min	71 (42, 132)	67 (44, 128)	79 (38, 140)	>0.99
Door to CT time (min), median (IQR), min	31 (20, 57)	32 (18, 52)	28 (22, 52)	0.82
Door to needle time, median (IQR), min	60 (46, 83)	61 (44, 82)	59 (53, 82)	0.92
Door to groin puncture time, median (IQR), min	155 (127, 220)	161 (129, 216)	139 (124, 224)	0.55
Door to recanalization time, median (IQR), min	277 (198, 355)	257 (203, 364)	294 (194, 325)	>0.99
Groin puncture to recanalization time, median (IQR), min	29 (23, 37)	25 (19, 31)	35 (30, 65)	0.015
mTICI score, *n* (%)				
2b	30 (29)	21 (28)	9 (30)	0.16
2c	20 (19)	11 (15)	9 (30)	
3	54 (52)	42 (57)	12 (40)	
Hemorrhagic transformation, *n* (%)	48 (46)	33 (45)	15 (50)	0.62
Type of hemorrhagic transformation, if present, *n* (%)				
HI1	9 (8.7)	7 (9.5)	2 (6.7)	0.32
HI2	17 (16)	11 (15)	6 (20)	
PH1	13 (13)	11 (15)	2 (6.7)	
PH2	9 (8.7)	4 (5.4)	5 (17)	
Discharge NIHSS score, median (IQR)	4 (2, 9)	3 (1, 6)	9 (4, 12)	<0.001
mRS score at 90 days, median (IQR)	2.00 (1.00, 4.00)	2.00 (1.00, 3.75)	4.00 (2.25, 6.00)	<0.001

Abbreviations: ASPECTS, Alberta Stroke Program Early Computed Tomograpy Score; CBV, Cerebral Blood Volume; CT, computed tomography; IQR, interquartile range; IVT, intravenous thrombolysis; mRS, modified Rankin Scale; MT, mechanical thrombectomy; mTICI, modified Thrombolysis in Cerebral Infarction; NIHSS, National Institutes of Health Stroke Scale; PVT, prolonged venous transit; rCBF, relative cerebral blood flow.

^a^ Wilcoxon rank sum test; Pearson's Chi‐squared test; Fisher's exact test.

Interventional data revealed a longer median groin puncture to recanalization time in the PVT+ group (35 [IQR 30–65] min) compared to the PVT− group (25 [IQR 19–31] min; *p* = 0.015). However, other procedural times, such as door‐to‐groin puncture and door‐to‐recanalization times, were similar between groups.

### Clinical outcomes

**FIGURE 3:**
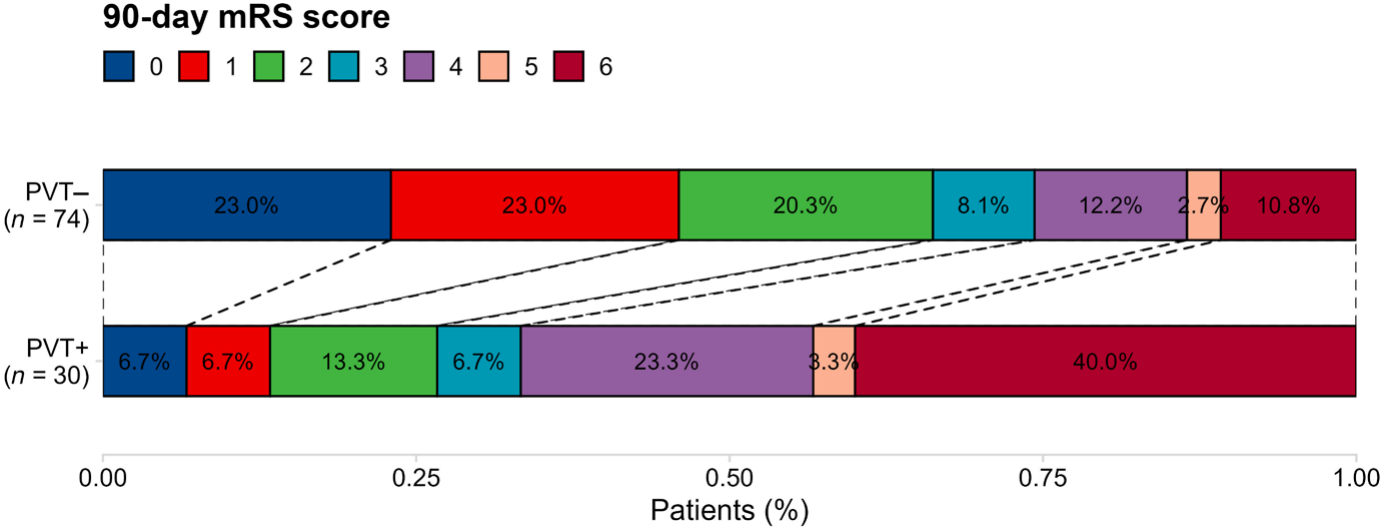
Distribution of 90‐Day modified Rankin Scale (mRS) scores among the patients with prolonged venous transit PVT (PVT+) and those without PVT (PVT–).

The primary outcome of excellent clinical recovery (90‐day mRS score 0–1) was achieved in 38 patients (37%). PVT positivity was associated with a significantly lower likelihood of excellent recovery (11% vs. 39%; *p* < 0.001). The PVT+ group had a higher median discharge NIHSS score (9 [IQR 4–12]) compared to the PVT− group (3 [IQR 1–6]; *p* < 0.001), and a higher median 90‐day mRS score (4 [IQR 2.25–6.00] vs. 2 [IQR 1.00–3.75]; *p* < 0.001 [Figure 3]).

### Logistic regression analysis of 90‐day excellent functional outcome

In the multivariable logistic regression model, PVT positivity was independently associated with lower likelihood of excellent clinical recovery at 90 days (OR 0.11, 95% CI 0.02–0.48; *p* = 0.006). Other factors associated with poor outcomes included higher admission NIHSS score (OR 0.83, 95% CI 0.73–0.92; *p* = 0.001). Variables such as age, hypertension, diabetes, and final mTICI score were not significantly associated with the primary outcome after adjusting for confounders (Table 3).

**TABLE 3:** Multivariable regression for outcome of excellent clinical outcome at 90 days (modified Rankin Scale score 0–1) in patients with successfully reperfused acute ischemic stroke due to anterior circulation large‐vessel occlusion.

Variable	mRS2‐6	mRS0‐1	Univariable Model		Multivariable Model	
	*N* = 66	*N* = 38	OR (95% CI)	*p*	OR (95% CI)	*p* value
Age, median (IQR)	72 (64, 82)	67 (53, 74)	0.97 (0.95 to 1.00)	0.054	0.98 (0.94 to 1.01)	0.19
Sex, *n* (%)						
Female	37 (56)	21 (55)	—			
Male	29 (44)	17 (45)	1.03 (0.46 to 2.31)	0.94		
Occlusion segment, *n* (%)						
ICA	5 (7.6)	3 (7.9)	—			
M1	49 (74)	27 (71)	0.92 (0.21 to 4.75)	0.91		
Poximal‐M2	12 (18)	8 (21)	1.11 (0.21 to 6.67)	0.9		
Smoking status, *n* (%)	34 (52)	20 (53)	1.01 (0.45 to 2.27)	0.97		
Alcohol use, *n* (%)	18 (28)	14 (37)	1.52 (0.64 to 3.59)	0.33		
Hypertension, *n* (%)	57 (86)	26 (68)	0.34 (0.12 to 0.91)	0.032	0.53 (0.15 to 1.89)	0.33
Dyslipidemia, *n* (%)	35 (53)	20 (53)	0.98 (0.44 to 2.20)	0.97		
Diabetes, *n* (%)	22 (33)	7 (18)	0.45 (0.16 to 1.14)	0.11		
Heart disease, *n* (%)	34 (52)	18 (47)	0.85 (0.38 to 1.88)	0.68		
Atrial fibrillation, *n* (%)	26 (39)	18 (47)	1.38 (0.62 to 3.11)	0.43		
History of stroke/TIA, *n* (%)	11 (17)	6 (16)	0.94 (0.30 to 2.71)	0.91		
Chronic kidney disease, *n* (%)	11 (17)	9 (24)	1.55 (0.57 to 4.18)	0.38	0.89 (0.22 to 3.46)	0.87
Sleep apnea, *n* (%)	6 (9.1)	5 (13)	1.52 (0.41 to 5.40)	0.52		
Admission NIHSS score, median (IQR)	17 (13, 20)	12 (8, 14)	0.84 (0.76 to 0.91)	<0.001	0.83 (0.73 to 0.92)	**0.001**
Premorbid mRS score, *n* (%)						
0	54 (82)	29 (78)	—			
1	12 (18)	8 (22)	1.24 (0.44 to 3.35)	0.67		
Occlusion laterality, *n* (%)						
Left	29 (49)	16 (52)	—			
Right	30 (51)	15 (48)	0.91 (0.38 to 2.17)	0.82		
ASPECTS, median (IQR)	9.00 (7.00, 10.00)	10.00 (8.00, 10.00)	1.15 (0.93 to 1.48)	0.23		
rCBF <20% volume, median (IQR), mL	0 (0, 11)	0 (0, 5)	0.97 (0.93 to 1.00)	0.1	0.97 (0.91 to 1.03)	0.38
Tmax >6 s volume, median (IQR), mL	119 (76, 167)	104 (66, 141)	1.00 (0.99 to 1.00)	0.18	1.00 (0.99 to 1.01)	0.47
Mismatch ratio, median (IQR)	3.8 (2.8, 7.1)	4.9 (3.0, 8.7)	1.06 (0.98 to 1.18)	0.17		
Hypoperfusion intensity ratio, median (IQR)	0.40 (0.20, 0.60)	0.40 (0.20, 0.50)	0.36 (0.06 to 2.21)	0.27		
CBV index, median (IQR)	0.80 (0.70, 0.90)	0.80 (0.73, 0.90)	5.78 (0.39 to 98.6)	0.21		
PVT, *n* (%)	26 (39)	4 (11)	0.18 (0.05 to 0.52)	0.004	0.11 (0.02 to 0.48)	**0.006**
Tan score (0–3), median (IQR)	2.00 (1.00, 2.00)	2.00 (1.00, 2.00)	1.04 (0.64 to 1.70)	0.87	0.50 (0.21 to 1.12)	0.1
IVT administered, *n* (%)	19 (29)	13 (34)	1.29 (0.54 to 3.02)	0.56	0.83 (0.24 to 2.69)	0.75
MT attempted, *n* (%)	66 (100)	38 (100)				
Symptom onset to door time, median (IQR), min	75 (31, 135)	66 (49, 125)	1.00 (1.00 to 1.00)	0.74		
Door to CT time, median (IQR), min	31 (22, 54)	31 (17, 60)	1.00 (1.00 to 1.01)	0.39		
Door to needle time, median (IQR), min	59 (40, 76)	68 (47, 93)	1.00 (1.00 to 1.00)	0.98		
Door to groin puncture time, median (IQR), min	144 (118, 203)	183 (141, 272)	1.01 (1.00 to 1.01)	0.14		
Door to recanalization time, median (IQR), min	277 (186, 332)	275 (224, 533)	1.00 (1.00 to 1.00)	0.76		
Groin puncture to recanalization time, median (IQR), min	30 (24, 39)	25 (22, 33)	1.01 (0.99 to 1.04)	0.47		
mTICI score, *n* (%)						
2b	24 (36)	6 (16)	—		—	
2c	11 (17)	9 (24)	3.27 (0.95 to 12.1)	0.064	4.71 (0.85 to 29.7)	0.083
3	31 (47)	23 (61)	2.97 (1.09 to 9.07)	0.041	3.40 (0.96 to 13.8)	0.068

Abbreviations: ASPECTS, Alberta Stroke Program Early Computed Tomograpy Score; CBV, Cerebral Blood Volume; CT, computed tomograpy; ICA, internal carotid artery; IQR, interquartile range; IVT, intravenous thrombolysis; mRS, modified Rankin Scale; MT, mechanical thrombectomy; mTICI, modified Thrombolysis in Cerebral Infarction; NIHSS, National Institutes of Health Stroke Scale; PVT, prolonged venous transit; rCBF, relative cerebral blood flow; TIA, transient ischemic attack.

### Ordinal regression analysis of 90‐day mRS score

Ordinal logistic regression showed that PVT positivity was independently associated with worse 90‐day mRS scores (adjusted OR 5.47, 95% CI 2.13 to 14.70; *p* < 0.001). Age (adjusted OR per year increase 1.05; 95% CI 1.02 to 1.08; *p* < 0.001) and admission NIHSS score (OR 1.12, 95% CI 1.04 to 1.20; *p* = 0.001) were also significant predictors (Table S1).

## DISCUSSION

In this study, we found that PVT was significantly associated with a lower likelihood of achieving excellent functional recovery (mRS score 0–1) at 90 days following successful reperfusion in patients with AIS‐LVO. Specifically, 11% of PVT+ patients achieved excellent recovery compared to 39% of PVT– patients (*p* < 0.001). Notably, although the median admission NIHSS score was slightly higher in PVT+ patients (16 [IQR 14–19]) compared to PVT– patients (14 [IQR 10–17]; *p* = 0.038), PVT remained independently associated with excellent functional outcome after adjusting for NIHSS score (adjusted OR 0.11, 95% CI 0.02–0.48; *p* = 0.006). This indicates that the association between PVT and outcome was not solely due to differences in initial stroke severity and suggests that PVT provides additional prognostic information beyond admission NIHSS score.

Our findings suggest that PVT positivity reflects complex pathophysiological interactions contributing to poorer outcomes in AIS‐LVO. PVT positivity is a surrogate for obstruction of VO through ischemic brain tissue, and increased resistance within the venous drainage system due to elevated interstitial pressure from tissue edema [32]. In contrast, PVT− patients may have more efficient blood transit through ischemic tissue, enhancing thrombolysis and preventing distal vessel occlusion after reperfusion [33, 34], which contributes to better outcomes [35, 36].

In this study, patients in the PVT+ group demonstrated a longer median puncture‐to‐recanalization time compared with those in the PVT− group (35 [IQR 30–65] min vs. 25 [IQR 19–31] min; *p* = 0.015). This may be attributable to several factors. Impaired VO may increase interstitial pressure and cerebral edema, creating additional procedural challenges, including more difficult catheter navigation [7, 9, 10, 11]. Furthermore, PVT+ patients may present with larger thrombus burdens or more complex occlusions due to suboptimal collateral circulation, necessitating a more prolonged and intricate MT process. Additionally, our analysis revealed no significant difference in collateral status between PVT+ and PVT− patients, with both groups having a median Tan score of 2.00 (IQR 1.00–2.00) and a *p* value of 0.089. This suggests that the observed association between PVT and functional outcomes is independent of arterial collateral circulation [7].

Our findings reinforce the growing recognition of VO as a critical determinant of functional outcomes in stroke patients [17, 32, 37]. While current reperfusion therapies primarily focus on restoring arterial blood flow, our data suggest that venous parameters, such as PVT, could provide valuable prognostic information in AIS‐LVO, thus our study suggests that incorporating PVT into routine imaging assessments as a surrogate for impaired VO could aid clinicians in refining post‐thrombectomy management and optimizing rehabilitation strategies.

Our study has potential clinical implications because the ease of PVT assessment using CTP‐derived Tmax maps provides a practical advantage. PVT is a simple, binary marker that can be readily interpreted, even by clinicians without extensive neuroradiological expertise. This simplicity contrasts with more complex venous scoring systems, such as the cortical vein opacification score, which requires detailed evaluation of multiple venous structures [38, 39].

Our results align with previous studies highlighting the critical role of VO in ischemic brain tissue microperfusion and its impact on clinical outcomes. Heitkamp et al. [33] and Faizy et al. [8, 40] identified unfavorable VO as a significant predictor of poor functional outcomes despite achieving successful reperfusion. Similarly, Adusumilli et al. [9] demonstrated that a favorable comprehensive venous profile is strongly associated with functional independence and excellent outcomes post‐thrombectomy. Moreover, Faizy et al. highlighted a strong correlation between favorable VO and robust tissue‐level collaterals, as indicated by a hypoperfusion intensity ratio of less than 0.4, which is linked to better functional recovery. Our findings are consistent with these studies, further supporting the notion that VO plays a pivotal role in determining clinical outcomes in AIS‐LVO patients. Additionally, the findings of this study align with previous research on PVT. For instance, a recent study reported a significant association between PVT positivity and increased 90‐day mortality, despite successful reperfusion (OR 1.22, 95% CI 1.02–1.46; *p* = 0.03) [41]. Another study found that PVT positivity was associated with decreased likelihood of achieving favorable clinical outcomes (adjusted OR 0.23, 95% CI 0.07–0.81; *p* = 0.02) [38]. In addition, PVT positivity has been correlated with poorer neurological function, including unfavorable functional recovery (*r* = 0.27; *p* = 0.002) and increased mortality (*r* = 0.26; *p* = 0.002) [42].

Our study has several limitations that should be considered. First, the retrospective design introduces inherent limitations such as selection bias and confounding factors. Second, the availability of CTP may be restricted in smaller centers and rural areas, which could affect the generalizability of our results. Third, PVT is influenced by multiple factors, including blood volume effects on blood flow, variations in drainage patterns from non‐affected regions, and imaging protocol specifics, such as contrast injection rate and bolus timing. Fourth, the PVT assessment in our study was limited to two representative locations for simplicity, which may reduce its comprehensive applicability and make it vulnerable to local pathologies, such as thrombosis. Future research should prioritize multicenter, prospective studies to further elucidate the association between VO profiles and stroke outcomes and to evaluate the generalizability of our findings across different populations and healthcare settings.

In conclusion, PVT, as assessed by CTP using Tmax ≥10 s in the SSS and/or torcula, was significantly associated with lower odds of excellent functional outcome (mRS score 0–1 at 90 days) in patients with AIS‐LVO of the anterior circulation, despite successful reperfusion. These findings highlight the potential role of pretreatment VO parameters, such as PVT, in prognostication and management decisions following MT. Future studies should focus on validating PVT as a prognostic biomarker in larger, multicenter, prospective cohorts to confirm its utility and generalizability in clinical practice.

## CONFLICTS OF INTEREST STATEMENT

The authors declare no conflicts of interest.

## Supporting information


Table S1.

## Data Availability

The data that support the findings of this study are available on request from the corresponding author. The data are not publicly available due to privacy or ethical restrictions.
