# Supplementary material for: Prolonged venous transit is associated with lower odds of excellent recovery after reperfusion in anterior large‐vessel occlusion stroke
Source: Eur J Neurol. 2024 Dec 2;32(1):e16563. doi: 10.1111/ene.16563 (PMC11609734; doi:10.1111/ene.16563)
Supplement: Supplementary file 1 — Table S1. [file ENE-32-e16563-s001.docx]

**Supplementary Table 1**. Multivariable Ordinal Regression for Outcome of mRS score at 90 Days in Patients With Successfully Reperfused AIS‐LVO

| **Variable** | **Univariable Model** | | | **Multivariable Model** | | |
| --- | --- | --- | --- | --- | --- | --- |
|  | **OR***^1^* | **95% CI***^1^* | **P** | **OR***^1^* | **95% CI***^1^* | **P** |
| Age | 1.04 | 1.02, 1.07 | **<0.001** | 1.05 | 1.02, 1.08 | **<0.001** |
| Sex |  |  |  |  |  |  |
| Female | — | — |  |  |  |  |
| Male | 0.97 | 0.49, 1.93 | 0.93 |  |  |  |
| Occlusion Segment |  |  |  |  |  |  |
| ICA | — | — |  |  |  |  |
| M1 | 1.56 | 0.48, 5.18 | 0.46 |  |  |  |
| Poximal-M2 | 1.31 | 0.34, 5.15 | 0.7 |  |  |  |
| Smoking Status | 1.42 | 0.72, 2.82 | 0.32 |  |  |  |
| Alcohol Use | 0.56 | 0.27, 1.17 | 0.13 |  |  |  |
| Hypertension | 2.1 | 0.92, 4.88 | 0.08 | 1.02 | 0.41, 2.58 | >0.9 |
| Dyslipidemia | 1.02 | 0.51, 2.00 | 0.97 |  |  |  |
| Diabetes | 2.32 | 1.08, 5.08 | **0.033** | 1.74 | 0.70, 4.45 | 0.2 |
| Heart Disease | 1.47 | 0.75, 2.91 | 0.27 |  |  |  |
| Atrial Fibrillation | 1.13 | 0.57, 2.25 | 0.72 |  |  |  |
| History of Stroke/TIA | 1.16 | 0.47, 2.89 | 0.75 |  |  |  |
| Chronic Kidney Disease | 0.62 | 0.26, 1.48 | 0.28 | 0.7 | 0.27, 1.80 | 0.5 |
| Sleep Apnea | 0.95 | 0.30, 3.07 | 0.93 |  |  |  |
| Admission NIHSS Score | 1.14 | 1.08, 1.21 | **<0.001** | 1.12 | 1.04, 1.20 | **0.001** |
| Premorbid Modified Rankin Scale |  |  |  |  |  |  |
| 0 | — | — |  |  |  |  |
| 1 | 0.74 | 0.32, 1.74 | 0.49 |  |  |  |
| Occlusion Laterality |  |  |  |  |  |  |
| left | — | — |  |  |  |  |
| right | 1.06 | 0.51, 2.20 | 0.88 |  |  |  |
| ASPECTS | 0.94 | 0.79, 1.11 | 0.48 |  |  |  |
| rCBF <20% volume (mL) | 1.02 | 1.0, 1.04 | 0.13 | 1.02 | 0.98, 1.06 | 0.3 |
| Tmax >6s volume (mL) | 1 | 1.00, 1.01 | 0.093 | 1 | 0.99, 1.00 | 0.4 |
| Prolonged venous transit | 5.36 | 2.42, 12.3 | **<0.001** | 5.47 | 2.13, 14.7 | **<0.001** |
| Mismatch Volume (mL) | 1 | 1.00, 1.01 | 0.15 |  |  |  |
| Hypoperfusion Intensity Ratio (HIR) | 3.36 | 0.75, 15.3 | 0.11 |  |  |  |
| Tan score (0-3) | 0.96 | 0.65, 1.42 | 0.84 | 1.49 | 0.87, 2.61 | 0.15 |
| IVT Administered | 0.68 | 0.33, 1.39 | 0.29 | 0.95 | 0.42, 2.17 | >0.9 |
| Symptom Onset to Door Time (mins) | 1 | 1.00, 1.00 | 0.34 |  |  |  |
| Door to CT Time (minutes) | 1 | 0.99, 1.00 | 0.27 |  |  |  |
| Door to Needle Time (minutes) | 1 | 1.00, 1.00 | 0.51 |  |  |  |
| Door to Groin Puncture Time (minutes) | 1 | 1.0, 1.00 | 0.59 |  |  |  |
| Door to Recanalization Time (mins) | 1 | 1.00, 1.00 | 0.42 |  |  |  |
| Groin Puncture to Recanalization Time (minutes) | 0.98 | 0.96, 1.01 | 0.17 |  |  |  |
| mTICI Score |  |  |  |  |  |  |
| 2b | — | — |  | — | — |  |
| 2c | 0.47 | 0.16, 1.32 | 0.15 | 0.48 | 0.16, 1.42 | 0.2 |
| 3 | 0.5 | 0.23, 1.08 | 0.079 | 0.55 | 0.23, 1.29 | 0.2 |
| *^1^* OR = Odds Ratio, CI = Confidence Interval | | | | | | |
